# Image-guided percutaneous renal cryoablation for stage 1 renal cell carcinoma with high surgical risk

**DOI:** 10.1186/s12957-015-0610-x

**Published:** 2015-06-10

**Authors:** Xiang Yan, Mingxin Zhang, Xiaoxiang Chen, Wang Wei, Rong Yang, Yang Yang, Weidong Gan, Hongqian Guo, Yang Wang, Guo-Ping Shi

**Affiliations:** Department of Urology, Affiliated Nanjing Drum Tower Hospital, Medical School of Nanjing University, Nanjing, China; Department of Radiology, Affiliated Nanjing Drum Tower Hospital, Medical School of Nanjing University, Nanjing, China; Medical School of Nanjing University, Nanjing, China; Department of Medicine, Brigham and Women’s Hospital, Harvard Medical School, Boston, USA

**Keywords:** Cryoablation, Renal cell carcinoma, Local anesthesia

## Abstract

**Background:**

This study was undertaken to evaluate the feasibility, safety, and therapeutic effects of percutaneous renal cryoablation under local anesthesia with conscious sedation for patients who have unresectable stage 1 (T1NoMo) renal cell carcinoma (RCC) in high surgical risk.

**Methods:**

Eighteen patients who were not candidates for surgery underwent primary cryosurgery guided by gray-scale ultrasound. Contrast-enhanced ultrasonography (CEUS) and contrast-enhanced computed tomography (CT) were performed to evaluate treatment at completion.

**Results:**

The mean follow-up period was 26.8 months (range, 12–56 months). All tumors were biopsied before cryosurgery. Seventeen tumors remained free of enhancement during follow-up period. No major complications associated with cryoablation procedures were found though two instances of subcapsular hematomas, one of retroperitoneal errhysis and one of nausea, were seen after cryoablation. One patient had a local recurrence of tumor and received additional cryoablation. Local tumor control rate was 100 % of T1NoMo tumors including the recurrence case who underwent additional cryoablation.

**Conclusions:**

Percutaneous cryoablation can be recommended as a feasible, safe, and promising therapy for the treatment of renal tumor, especially those unresectable stage 1 RCC, with a low risk of complications.

## Background

By reason of the incidental detection of small and asymptomatic masses with the widespread use of imaging techniques, the incidence of renal cell carcinoma has increased by severalfold during the past two decades [1, 2]. Despite that less invasive surgical procedures such as laparoscopic partial nephrectomy [3] and laparoscopic renal cryoablation [4] have been performed in select patients, the demand for further reductions in therapy and general anesthesia related to morbidity is desirable. However, particularly in high-risk patients, an even less invasive treatment such as percutaneous renal cryoablation would be feasible. For some high-surgical-risk patients, we were able to perform percutaneous cryoablation under local anesthesia with conscious sedation for unresectable stage 1 (T1NoMo) renal cell carcinoma (RCC). In our study, we evaluated the feasibility, safety, and therapeutic effects of percutaneous renal cryoablation for patients with high surgical risk after a 2-year mean follow-up.

## Methods

### Patients and eligibility criteria

From April 2009 to July 2013, 18 selective patients with T1NoMo RCC underwent percutaneous renal cryoablation. All patients gave their informed consent and Nanjing Drum Tower Hospital's ethics committee approved the study protocol. Twelve men and six women with mean age of 62 years (range, 41–89 years) were included in this study. A total of ten tumors were found in the right kidney and eight in the left. The mean tumor size was 3.3 ± 1.2 cm (range, 1.4–4.6 cm). All tumors were exophytic. All of 18 patients had T1NoMo tumors according to the TNM staging system. All patients were not candidates for surgery due to the fact that they are receiving treatment for other cancers (*n* = 3), age >85 years old and refusal of surgical treatment (*n* = 4), hemophilia (*n* = 1), coronary heart disease (*n* = 6), end-stage renal disease receiving hemodialysis (*n* = 2), dilated cardiomyopathy (DCM) with heart failure (*n* = 2). Patients’ characteristics and eligibility criteria are summarized in Table 1. The diagnosis of RCC was based on the results of needle biopsy before cryosurgery.

**Table 1:** Characteristics of patients and tumors for percutaneous cryoablation

Patient characteristics	Result
Patients (*n*)	18
Mean age (range)	62 (41–89)
Sex (*n* male/female)	12/6
Body mass index (kg/m^2^)	22.2 ± 3.7
The combined surgical risk factors:	
Receiving treatment for other cancers	3
Age >85 years old and refusal of surgery	4
Hemophilia	1
Coronary heart disease	6
End-stage renal disease receiving hemodialysis	2
Dilated cardiomyopathy with heart failure	2
Mean follow-up (range), months	26.8 (12–56)
Mean tumor size (range), cm	3.3 (1.4–4.6)
Tumor location (right/left)	10/8
Pathology:	
Clear cell RCC	14
Papillary RCC	3
Chromophobe RCC	1
Operative time (min)	67 ± 18
Complication	
Nausea	3
Pain	4
Subcapsular hemorrhage	1
Retroperitoneal errhysis	1

### Percutaneous renal cryoablation procedures

All cryoablation procedures were performed using the Cryo-Hit System (Galil Medical Ltd, Israel) by a single urologist. All patients were placed in a modified lateral position after induction with local anesthesia. Under ultrasound (US) guidance, 1.47 mm 17G IceSeed cryoneedles were placed into the renal tumor. The number of cryoneedles varied with the size of tumor. Freezing with argon gas to < −40 °C was initiated and monitored using the temperature-monitoring probes, and US guidance, which revealed an acoustic shadow as the ice ball formed. After maintaining −40 °C or the lowest temperature below −25 °C for 10 to 15 min, a passive thaw was initiated until the temperature reached a plateau. At this point, active thawing with helium was started. Two cycles of rapid freeze–thaw was carried out, ensuring the temperature in the renal tumor and just outside it was below a therapeutic value of −40 °C. At the end of the procedure, the needles and probes were removed and pressure applied to location for 10 min to reduce bruising.

### Evaluation at follow-up

Therapeutic effects were evaluated by contrast-enhanced ultrasonography (CEUS) and contrast-enhanced computed tomography (CT) 1 month after percutaneous renal cryoablation. Subsequent CEUS and contrast-enhanced CT assessment were performed after cryoablation for additional follow-up imaging at 3, 6, and every 6 months thereafter. All lesions that showed the non-enhancing ablation zone on CEUS and contrast-enhanced CT was considered to be a successful cryoablation. The second cryoablation treatment was performed additionally when an enhancing area of cancer tissue was still present. Renal function of each patient was evaluated with serum creatinine and the glomerular filtration rate (GFR = 175 × (serum creatinine) × 11.54(age) × 0.203 × (0.742 if female) × (1.210 if black)) [5].

## Results

### Procedures and therapeutic effects

**Fig. 1:**
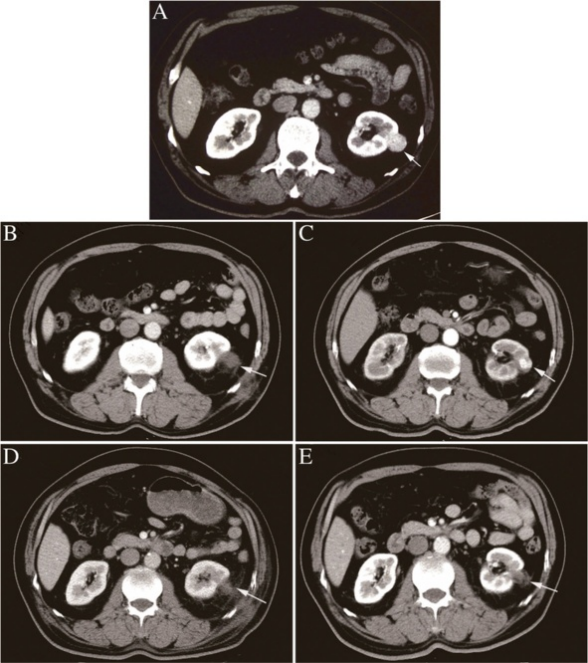
Sequential contrast-enhanced CT images of the ablation area of renal cell carcinoma (RCC) measuring 2.8 cm observed for the longest period with two cryoablations. **a** Contrast-enhanced CT before cryotherapy. **b** Contrast-enhanced CT 1 month after stage 1 cryotherapy. **c** Contrast-enhanced CT 12 months after stage 1 cryotherapy. **d** Contrast-enhanced CT 1 month after stage 2 cryotherapy. **e** Contrast-enhanced CT 12 months after stage 2 cryotherapy

**Fig. 2:**
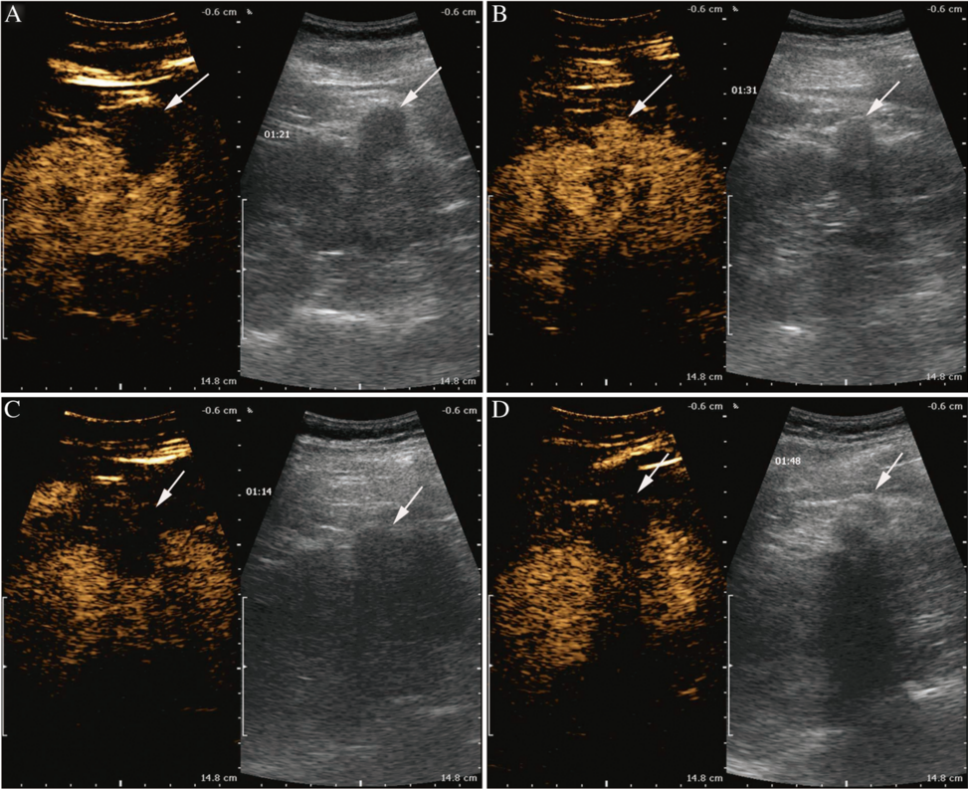
Sequential contrast-enhanced ultrasound images of the ablation area of renal cell carcinoma (RCC) observed for the period with two cryoablations. **a** Contrast-enhanced ultrasound 1 month after stage 1 cryotherapy. **b** Contrast-enhanced ultrasound 12 months after stage 1 cryotherapy. **c** Contrast-enhanced ultrasound 1 month after stage 2 cryotherapy. **d** Contrast-enhanced ultrasound 12 months after stage 2 cryotherapy

Cryoablation proved to be technically successful in all patients under local anesthesia with conscious sedation, and tumors were considered completely ablated after at least two follow-up sessions. Patients could tolerate renal cryoablation successfully with minimal requirements for pain medication and without the need for blood transfusions. The mean duration of cryosurgery was 59.7 ± 11.6 min. The mean length of stay in the hospital for the patients after cryoablation was 3.2 ± 1.2 days. One patient had a recurrence of tumor 1 year later and accepted additional cryoablation. As the image showed a sequential image at a 2-year follow-up, it demonstrated the size reduction and disappearance of tumor’s enhancement of the ablated lesion completely for the longest time after two cryoablations (Figs. 1 and 2). Figure 1a showed contrast-enhanced CT obtained before cryosurgery showed enhancement of the tumor. Figures 1b and 2a demonstrated absence of enhancement of the ablated tumor 1 month after stage 1 cryotherapy. Figures 1c and 2b showed an enhancing area of cancer tissue was still present 12 months after stage 1 cryotherapy. The patient was then scheduled to have a stage 2 cryotherapy. Figures 1d and 2c demonstrated no enhancement of the ablated lesions 1 month after stage 2 cryotherapy. Figures 1e and 2d demonstrated no growth or enhancement of those lesions 12 months after stage 2 cryotherapy.

### Complications

No major complications were found during the procedures though intraoperative complications such as nausea and pain, but nausea and pain are within tolerable limits in all patients. As postoperative complications, one patient had subcapsular hemorrhage and another one had retroperitoneal errhysis. No patient required dialysis, either temporary or permanent. The pre- and post-cryoablation mean serum creatinine levels were 1.2 ± 0.7 mg/dL and 1.3 ± 0.6 mg/dL, respectively. Also, no significant differences were found in the pre- and post-cryoablation mean glomerular filtration rate (49.7 ± 22.1 mL/min and 50.1 ± 21.7 mL/min, respectively).

### Follow-up findings

Seventeen of eighteen tumors were confirmed completely ablated and had no growth or enhancement during the mean follow-up period of 26.8 months. All tumors had a significant reduction from 3.3 ± 1.2 cm to 2.7 ± 1.1 cm (*P* < 0.05) observed at the last follow-up examination after cryoablation. Only one tumor measuring 2.8 cm was confirmed incompletely ablated by contrast-enhanced CT scan 12 months after the procedure and underwent re-cryoablation. No metastases of any tumors were found during the follow-up period. The recurrence rate of RCC after successful cryoablation was 5.5 % (1/18). The local tumor control rate was 100 % including the recurrence case that underwent re-cryoablation.

## Discussion

The role of various forms of minimally invasive therapies has been greatly expanded to the previous standard of open radical nephrectomy. Although partial nephrectomy remains the reference standard, ablative techniques are increasingly applied in the management of small renal tumors with the long-term results emerging [6]. Among ablative modalities, cryotherapy is the most widely used and accepted, especially for older patients in high risk [7], and has shown to correlate with minimal complications and good intermediate-term oncologic outcomes [8]. The application of cryoablation is to ablate solid tumors found in the lung, liver, breast, kidney, and prostate. Prostate and renal cryoablation are the most common. Although sometimes cryoablation is applied through laparoscopic or open surgical approaches, most often cryoablation is performed percutaneously. The advantages of percutaneous renal cryoablation include a less-invasive procedure, shorter hospitalization, excellent ice ball monitoring with ultrasound, less requirement for pain medication, and lower risk of metastatic progression [9–11]. Most of all, it can be performed repeatedly [12]. Compared to other thermal ablation, cryoablation offers real-time, visual feedback of the ice ball and it is nearly painless during the procedure, eliminating the need of general anesthesia or deep sedation. Percutaneous renal cryoablation can be performed by ultrasound, CT, or MRI guidance. Ultrasound is convenient and sufficient for precise needle placement, and what is more, it allows real-time guidance. The selected patients in this study are all not candidates for surgical procedure because they are not could not tolerate the postoperative pain and complications which general anesthesia brings.

As a matter of fact, the most common sequelae are self-limited pain and paresthesia at the sites of probe insertion related to cryoablation. Other complications that are rare include hemorrhage, infection, visceral injury, pneumothorax, and delayed UPJ obstruction. Mild perinephric hematoma, myoglobinaemia, and transfusion have been reported [13–15], but in most cases, those complications could be managed rightly without operative procedure. By placing the cryoprobe perpendicularly into the kidney, carefully maintaining its position throughout the procedure, and deliberately removing the probe after complete thawing of renal tissue surrounding the cryoprobe, renal fracture and hemorrhage can be avoided [7]. Uzzo and Novick published a cumulative total of 155 (13.7 %) complications in 1129 procedures in a review of nephron-sparing surgical procedures during the past decade [16]. These complications include 14 deaths, 78 urinary fistulas, 3 splenic injuries, 19 infections or abscesses, 27 hemorrhages, and 18 patients had to receive postoperative dialysis [16]. The results of cryoablation in our study are superior to this standard, especially when one considers that many high-risk patients would not be candidates for surgical procedure. Atwell reported the durability of this treatment method with a low incidence of tumor recurrence beyond 3 months in a midterm follow-up of 93 tumors of percutaneous renal cryoablation [17]. Our recurrence rate of stage 1 RCC after successful cryoablation during a 2-year follow-up was 5.5 % (1/18). However, the repeated treatment was performed later resulting in the elimination of tumor enhancement. Although the local ablated tumor control was 100 % of patients including the recurrence case that received additional cryoablation at 2-year follow-up, we still need further investigation of the long-term safety and efficacy in tumor control. Moreover, in order to strengthen the treatment, cryoablation can be combined with preoperative or postoperative radiation therapy and chemotherapy treatment, which can be in our follow-up study.

## Conclusions

In summary, the result of our study showed percutaneous renal cryoablation is recommended as a feasible, safe, and treatment modality for select patients with unresectable stage 1 RCC, especially those older ones with high surgical risk. Long-term efficacy will be determined by continued maturation of data.
